# Eagle’s Syndrome: A Case Report of a Unilateral Elongated Styloid Process

**DOI:** 10.7759/cureus.4430

**Published:** 2019-04-10

**Authors:** Aravind Warrier S, Nanthini KC, Subadra K, Dhivya M Harini

**Affiliations:** 1 Oral Medicine and Radiology, Sri Ramachandra Institute of Higher Education and Research, Chennai, IND; 2 Oral Medicine and Radiology, Adhiparasakthi Dental College and Hospital, Chennai, IND

**Keywords:** eagle’s syndrome, elongated styloid process, facial pain

## Abstract

When styloid process elongation or stylohyoid ligament calcification can lead to various symptoms, such as dysphagia, facial pain, globus sensation, and headache, it is termed Eagle’s syndrome. It may be unilateral or bilateral. Though the overall prevalence in adults is 4%, only 0.16% of patients are symptomatic. Since the symptoms mimic several other orofacial pains and neuralgia, the diagnosis must be made through a detailed history, clinical examination, and various imaging modalities. The case of facial pain in a 22-year-old female patient who was diagnosed to have a unilateral elongated styloid process is discussed in this paper.

## Introduction

The styloid process is a pointed, lean, bony projection situated at the front of the stylomastoid foramen and arises from the temporal bone [1]. In 1652, Pietro Marchetti was the first to describe an ossifying process of the stylohyoid ligament [2].Later, in 1937, an otorhinolaryngologist named Eagle first described a syndrome characterized by an elongated styloid process and pain in the cervicofacial region [3]. The prevalence is about 4% of the population with most of them being asymptomatic [4] and between 4% and 10% of the patients having an elongated styloid experience the symptoms [5]. Women are more frequently affected as compared to men and the average age of the patients presenting with symptoms is usually 40 years. This is attributed to the fact that as age advances, the elasticity of the soft tissues and the associated ligaments is lost, putting increased pressure on the adjoining hard tissues [6]. In most of the individuals, the normal styloid process length ranges between 2 cm and 3 cm, and it is considered elongated when it is longer than 3 cm [3].

The presenting symptoms include dull, aching pain on either side of the throat, difficulty in swallowing, foreign body sensation in the throat, pain in the facial region, and recurrent headache and vertigo [4]. Since these symptoms mimic many maxillofacial and oropharyngeal disorders and neuralgias, a thorough clinical history, examination, and radiological assessment are necessary for attaining a diagnosis. Here, we present one such case of Eagle’s syndrome in a young female patient and explain the diagnosis and successful management of the same.

## Case presentation

A 22-year-old female patient reported to our clinic and complained of pain in the right side of her face and neck for one year. The pain was spontaneous in nature, its intensity was dull to moderate, and it was of intermittent nature. The pain aggravated while opening the mouth or moving the head and neck from side to side. She also experienced pain during swallowing, with an associated foreign body sensation in the throat. Her medical history is noncontributory. She underwent surgical removal of impacted teeth one year before for the same complaint; however, she did not have any relief from symptoms.

**Figure 1:**
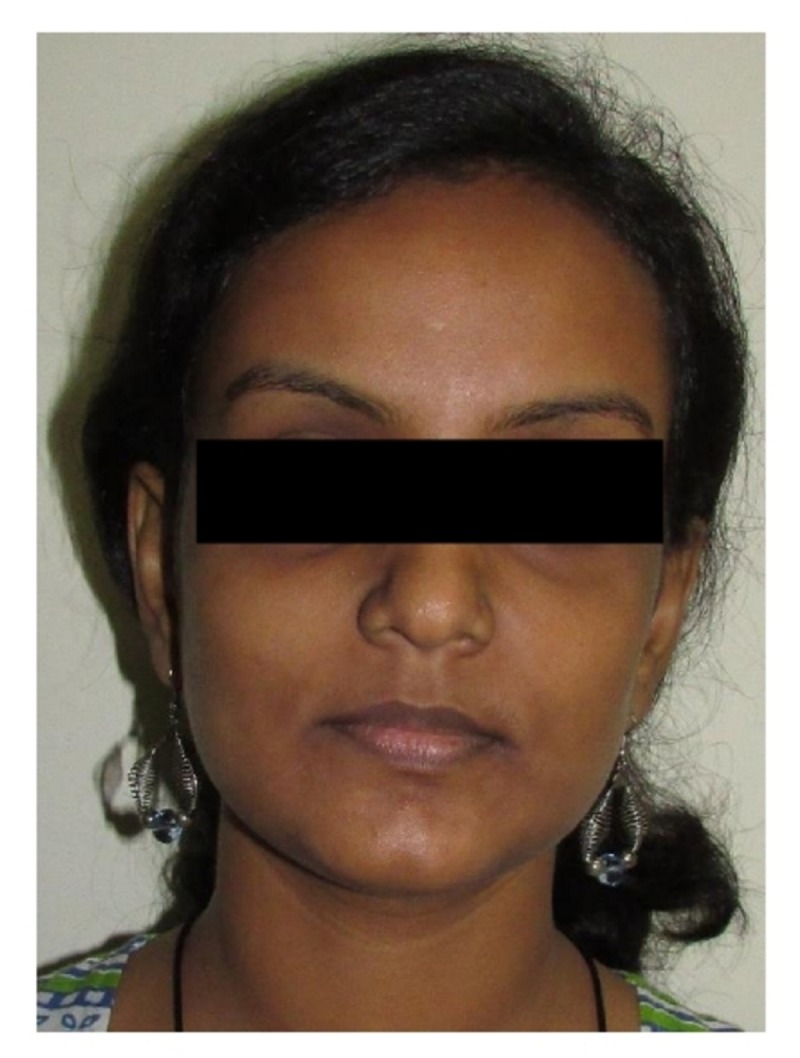
A 22-year-old female patient presented with pain in the right side of the face and neck, without any visible deformity

On an extraoral examination, the face was symmetrical (Figure 1). There was no palpable mass or tenderness in the involved region. There was no tenderness in the muscles of mastication during palpation. No tenderness was elicited in the temporomandibular joints during mandibular movements. On an intraoral examination, the patient experienced extreme tenderness on palpating the right tonsillar fossa. A bony mass was palpable in the same region.

**Figure 2:**
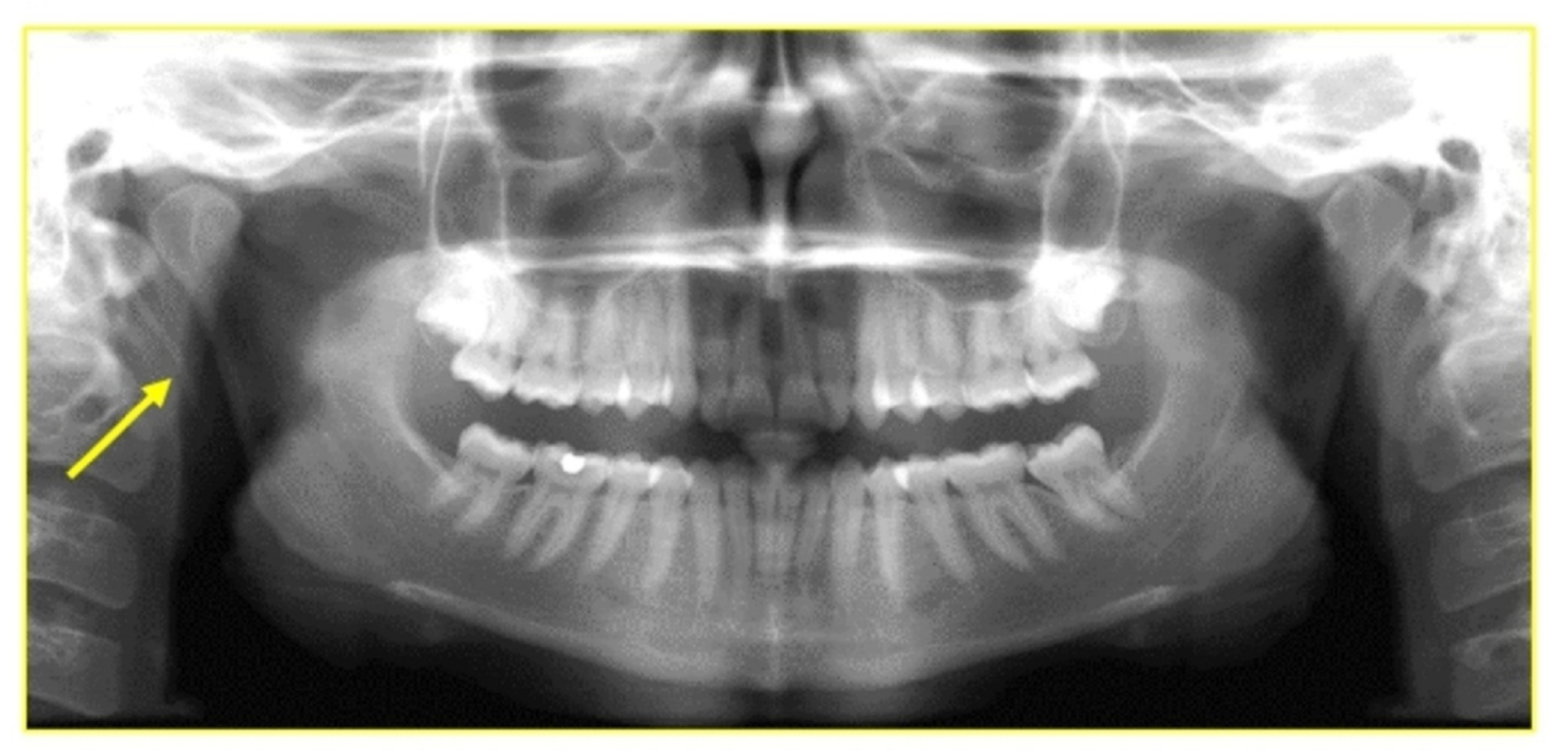
Panoramic radiograph showing the increased length of the styloid process (arrow) on the right side

**Figure 3:**
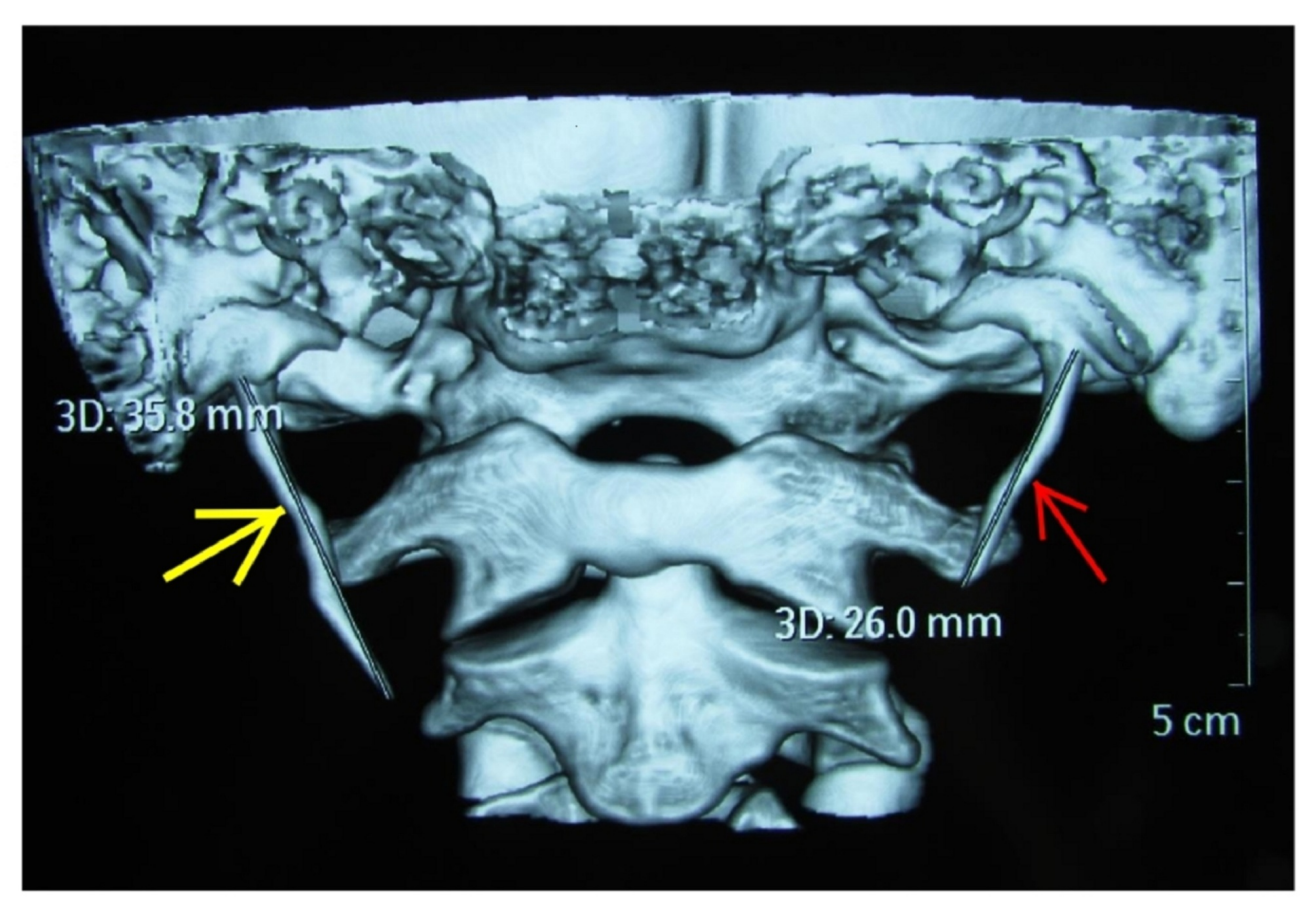
Computed tomography with three-dimensional reconstruction image revealing the length of the styloid process as 35.8 mm (yellow arrow) on the right and 26 mm (red arrow) on the left

An orthopantomogram was taken for the patient, and it revealed an increase in the length of the styloid process on the right side (Figure 2). This finding was further analyzed with computed tomography three-dimensional reconstruction imaging. Image analysis revealed an elongated right styloid process measuring 35.8 mm and the left side styloid process was 26 mm (within normal limits) (Figure 3). These findings led to the confirmatory diagnosis of Eagle’s syndrome.

The elongated styloid process on the right side was removed through the intraoral surgical approach at the level of the tonsillar fossa. The patient was free of symptoms one month after surgery, and after six months, the patient was completely asymptomatic.

## Discussion

The symptomatic calcification of the stylohyoid ligament complex or the elongation of the styloid process is termed Eagle’s syndrome [3]. Eagle reported around 200 cases over a period of 20 years and defined that the normal length of the styloid process is around 2.5 cm to 3 cm. He also noted that even a minor deviation of the styloid process toward the medial aspect can lead to symptoms of severe atypical facial pain [7]. Eagle classified two forms of the syndrome, namely, carotid artery and classic type. In the classic type, pain may occur after tonsillectomy where the scar tissue underneath the tonsillar fossa compresses the V, VII, IX, and X cranial nerves, causing discomfort and difficulty in swallowing and a feeling of having a foreign object in the throat. The carotid artery type has symptomatology characterized by headache and nerve problem due to the inflammation of the sympathetic nerve plexus [2]. In the present case, the patient had symptoms of the classic type.

The trademark of the syndrome is the archetypal mild and nagging pain caused by styloid process elongation, which worsens at the point of swallowing and it can be confirmed by palpating the tonsillar fossa [8]. The mean duration of symptoms is 14 months, the mean age of diagnosis is usually in the 3rd and 4th decades of life, with a greater female predilection and very rare occurrence in young patients [9]. The present case shows the rare occurrence of this syndrome in a young patient.

The pathogenesis is still in debate, and the major theories depend on embryology, heredity, granulation tissue proliferation following trauma, degenerative alterations, metaplasia, and nerve compression [10]. Also, the calcification patterns of this elongated stylohyoid complex play an important role in the pathogenesis [8]. The diagnostic workup for a patient assumed to have Eagle’s syndrome must include a complete history and a thorough examination of the head and neck clinically to rule out other differential diagnoses. Also, the symptoms can be reproduced by palpation over the stylohyoid complex cautiously. This will help in localization of the pain when the patient performs oral and cervical movements. The tip of the styloid process can be palpated at the level of the tonsillar fossa as a bony spicule, which is hard and, when palpated, can cause local tenderness and associated symptoms [11]. The definitive step to confirm the diagnosis is through specialized imaging. Numerous imaging modalities are in use for the investigation of the elongated styloid process, which includes orthopantomogram, Towne’s projection, lateral cephalogram, lateral oblique view of the mandible, anteroposterior skull radiographs, cone beam computed tomography (CT), and CT. In 1986, Langlais et al. [4] classified the radiographic features of the elongated styloid process based on its length and pattern of calcification. This helps every practitioner to describe the radiographic appearance of the elongated styloid process. Recent advances, such as three-dimensional CT, are being used by most practitioners as the radiological investigation of choice for the diagnosis, as they precisely measure the length, angulation, and calcification of the styloid process [12]. The various differential diagnoses include migraine, trigeminal, glossopharyngeal and other neuralgias, tonsillitis, otitis, psychosomatic, and inflammatory and neoplastic diseases of the orofacial region [8].

Conservative treatment options include non-steroidal anti-inflammatory drugs and injection of corticosteroids and local anesthetics into the tonsillar fossa. Surgical excision by the intraoral or extraoral approach has often been successful [9]. In the present case, an intraoral approach was used owing to its simple procedure, less operative time, and no extraoral scars.

## Conclusions

Unilateral elongation of the styloid process is usually a rare occurrence, and when this kind of symptom occurs in young patients, it makes for a diagnostic dilemma. It is mandatory for every dental specialist involved in the treatment of orofacial pain to be aware of various clinical presentations of Eagle’s syndrome and to include it in the differential diagnosis of such cases, to help in precise management.
